# Enhancing Nervous System Recovery through Neurobiologics, Neural Interface Training, and Neurorehabilitation

**DOI:** 10.3389/fnins.2016.00584

**Published:** 2016-12-27

**Authors:** Max O. Krucoff, Shervin Rahimpour, Marc W. Slutzky, V. Reggie Edgerton, Dennis A. Turner

**Affiliations:** ^1^Department of Neurosurgery, Duke University Medical CenterDurham, NC, USA; ^2^Department of Physiology, Feinberg School of Medicine, Northwestern UniversityChicago, IL, USA; ^3^Department of Neurology, Feinberg School of Medicine, Northwestern UniversityChicago, IL, USA; ^4^Department of Integrative Biology and Physiology, University of California, Los AngelesLos Angeles, CA, USA; ^5^Department of Neurobiology, Duke University Medical CenterDurham, NC, USA; ^6^Research and Surgery Services, Durham Veterans Affairs Medical CenterDurham, NC, USA

**Keywords:** neurorehabilitation, neural stimulation, brain-machine interface (BMI), neuroplasticity, neural regeneration, neural interface, neural repair, spinal cord stimulation

## Abstract

After an initial period of recovery, human neurological injury has long been thought to be static. In order to improve quality of life for those suffering from stroke, spinal cord injury, or traumatic brain injury, researchers have been working to restore the nervous system and reduce neurological deficits through a number of mechanisms. For example, neurobiologists have been identifying and manipulating components of the intra- and extracellular milieu to alter the regenerative potential of neurons, neuro-engineers have been producing brain-machine and neural interfaces that circumvent lesions to restore functionality, and neurorehabilitation experts have been developing new ways to revitalize the nervous system even in chronic disease. While each of these areas holds promise, their individual paths to clinical relevance remain difficult. Nonetheless, these methods are now able to synergistically enhance recovery of native motor function to levels which were previously believed to be impossible. Furthermore, such recovery can even persist after training, and for the first time there is evidence of functional axonal regrowth and rewiring in the central nervous system of animal models. To attain this type of regeneration, rehabilitation paradigms that pair cortically-based intent with activation of affected circuits and positive neurofeedback appear to be required—a phenomenon which raises new and far reaching questions about the underlying relationship between conscious action and neural repair. For this reason, we argue that multi-modal therapy will be necessary to facilitate a truly robust recovery, and that the success of investigational microscopic techniques may depend on their integration into macroscopic frameworks that include task-based neurorehabilitation. We further identify critical components of future neural repair strategies and explore the most updated knowledge, progress, and challenges in the fields of cellular neuronal repair, neural interfacing, and neurorehabilitation, all with the goal of better understanding neurological injury and how to improve recovery.

## Introduction

Historically, for patients suffering from spinal cord injury (SCI), stroke, or traumatic brain injury (TBI), the prognosis for recovery has been poor, and patients with more complete and chronic injuries have shown the least potential for improvement (Jennett et al., 1976; Waters et al., 1992, 1996; Curt et al., 2008; Perel et al., 2008; Steyerberg et al., 2008; Lloyd-Jones et al., 2010). Researchers have been dedicated to improving the quality of life for these patients in several ways, e.g., (1) biological manipulation of the cellular milieu to encourage neuronal repair and regeneration (Magavi et al., 2000; Chen et al., 2002; Lee et al., 2004; Freund et al., 2006; Benowitz and Yin, 2007; Park et al., 2008; Maier et al., 2009; de Lima et al., 2012b; Dachir et al., 2014; Li et al., 2015; Omura et al., 2015), (2) creation of neural- or brain-machine interfaces designed to circumvent lesions and restore functionality (Wolpaw and McFarland, 1994; Kennedy and Bakay, 1998; Leuthardt et al., 2004; Monfils et al., 2004; Hochberg et al., 2006, 2012; Moritz et al., 2008; O'Doherty et al., 2009; Ethier et al., 2012; Collinger et al., 2013; Guggenmos et al., 2013; Ifft et al., 2013; Memberg et al., 2014; Zimmermann and Jackson, 2014; Grahn et al., 2015; Jarosiewicz et al., 2015; Soekadar et al., 2015; Bouton et al., 2016; Capogrosso et al., 2016; Donati et al., 2016; Hotson et al., 2016; Rajangam et al., 2016; Vansteensel et al., 2016), and (3) new rehabilitation techniques that include electrical stimulation and pharmacological enhancement of spinal circuitry to stimulate recovery (Carhart et al., 2004; Levy et al., 2008, 2016; Dy et al., 2010; Harkema et al., 2011, 2012; Dominici et al., 2012; van den Brand et al., 2012; Gad et al., 2013b, 2015; Angeli et al., 2014; Gharabaghi et al., 2014a,c; Wahl et al., 2014; Gerasimenko et al., 2015b). Unfortunately, the path to clinical relevance for these individual approaches remains long, and each field tends to operate largely in its own sphere of influence. Nonetheless, there is now emerging evidence that these methods may synergistically enhance recovery of native motor function that can persist even after the training period and is beyond what was previously thought possible (van den Brand et al., 2012; Guggenmos et al., 2013; Angeli et al., 2014; Wahl et al., 2014; Gad et al., 2015; García-Alías et al., 2015). Some animal models are even displaying functional axonal regrowth, sprouting, and rewiring never seen before in the central nervous system (CNS) of mammals (Bregman et al., 1995; Chen et al., 2002; Liebscher et al., 2005; Freund et al., 2006; Maier et al., 2009; van den Brand et al., 2012; Wahl et al., 2014; García-Alías et al., 2015). Throughout much of this work, evidence is emerging that combinatorial therapy across fields may actually be necessary to achieve significant and lasting neurological repair (Wahl et al., 2014; Gad et al., 2015). This paper explores the state of the art in each of these disciplines, identifies essential components of rehabilitation strategies, and argues why synthesizing approaches across specialties will be essential to realizing clinical applicability.

## The biology of neurological injury

In order to understand how best to reverse or repair neurological injury, the mechanisms of cellular development and damage response must be appreciated. During maturation, young neurons of the CNS require activity (stimulated by purposeful actions like vision, walking, or hand function) and trophic factors to survive, grow, and prune (reviewed in Liu et al., 2011). Once mature, however, their axonal growth potential declines due to changes in intrinsic and extrinsic signaling factors, as well as established and stable synaptic fields. After lesioning, the distal portion of an axon undergoes Wallerian degeneration while the proximal portion seals the damaged membrane to form an end bulb (Schlaepfer and Bunge, 1973; Li and Raisman, 1995; Shetty and Turner, 1999; Hill et al., 2001; Fishman and Bittner, 2003). Eventually a growth cone is formed and injured corticospinal axons make an attempt to regrow; however, guidance cues are typically missing, and such efforts are therefore transient, abortive, and ultimately fail (Bernstein and Stelzner, 1983; Magavi et al., 2000; reviewed in Bulinski et al., 1998; Benowitz and Yin, 2007). This inability to regenerate is why injury to the CNS is so devastating and has been considered static once chronic.

After an insult, damaged CNS neurons continue a downward spiral of degeneration known as secondary injury. This includes an uncontrolled release of glutamate from presynaptic vesicles, loss of cell membrane potential, and damage to N-methyl-D-aspartate (NMDA) and a-amino-3-hydroxy-5-methyl-4-isoxazolepropionic acid (AMPA) receptors, all of which lead to overstimulation and increased neuronal death (Park et al., 1989; Goforth et al., 1999; Yurkewicz et al., 2005). Nitric oxide, triggered by the activation of NMDA receptors and intracellular calcium, interacts with stress-induced reactive oxygen species to cause DNA fragmentation, lipid peroxidation, and cellular death (reviewed in DeFina et al., 2009; Demirtas-Tatlidede et al., 2012; Villamar et al., 2012). Hemorrhage itself also exacerbates injury, as blood outside vessels releases excitatory amino acids, iron, and thrombin which induce further oxidative stress (Xi et al., 2006). The accumulation of excess intracellular zinc has too been shown to play a role in secondary injury by triggering neuronal death through intrinsic mechanisms such as 5′-adenosine monophosphate-activated protein kinase (AMPK) (Suh et al., 2000; Eom et al., 2016). These mechanisms of primary and secondary neurologic injury are summarized in Table 1.

**Table 1:** **Mechanisms of neurological injury**.

**Primary injury**	**Secondary injury**
Blunt trauma	Excitotoxicity (NMDA/AMPA—glutamate)
Penetrating trauma	Reactive O2 species (cytochrome c—caspase, GSH)
Necrosis/apoptosis	Nitric oxide (DNA fragmentation, lipid peroxidation
Degenerative disease	Zinc toxicity (PKC, NADPH, nNOS, PARP, AMPK)
Ischemia/stroke	Inflammation (BBB and CBF disruption, vasospasm)
Hemorrhage	Hemorrhage products (amino acids, iron, thrombin)
Infection	Cerebral edema (vasogenic, cytotoxic)
	Stroke sequelae (elevated ICP, edema, vasospasm)

*NMDA, N-methyl-D-aspartate; AMPA, α-amino-3-hydroxy-5-methyl-4-isoxazolepropionic acid; GSH, glutathione; DNA, deoxyribonucleic acid; PKC, protein kinase C; NADPH, nicotinamide adenine dinucleotide phosphate-oxidase; nNOS, neuronal nitric oxide synthase; PARP, poly adenosine diphosphate (ADP) ribose polymerase; AMPK, adenosine monophosphate (AMP)-activated protein kinase; BBB, blood brain barrier; CBF, cerebral blood flow; ICP, intracranial pressure*.

**Figure 1:**
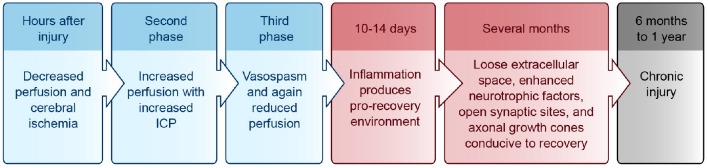
**Injury environment timeline**. Blue, acute phase; Red, subacute phase; Black, chronic phase. ICP, intracranial pressure.

Upon cell dissolution and fragmentation, inflammation is triggered, resulting in the clean-up of dead cells, disconnection of nonfunctional synapses, release of pro- and anti-inflammatory cytokines, and disruption of the blood-brain-barrier (BBB) (reviewed in Greve and Zink, 2009). This drives astrocytes and endothelial cells to produce more inflammatory mediators and impairs the brain's ability to manage its own perfusion status [i.e., autoregulate cerebral blood flow (CBF)]. During the first hours after injury, decreased perfusion and cerebral ischemia is seen, followed by a second phase of increased perfusion with increased intracranial pressure (ICP), and a final phase of vasospasm and again reduced perfusion (reviewed in Villamar et al., 2012). Until about 10–14 days after injury, inflammation helps to prime the extracellular milieu for subsequent axonal entry and re-innervation (Shetty and Turner, 1995). Resolution of acute inflammation is mediated by apoptosis of the inflammatory cells and endogenous anti-inflammatory mediators (reviewed in Villamar et al., 2012). A brief timeline of the injury environment is provided in Figure 1.

As a result of these inflammatory processes, cerebral edema tends to emerge during the secondary injury and can be either vasogenic or cytotoxic (Marmarou et al., 2006). Vasogenic edema results from vasodilation, increased permeability of the BBB, and accumulation of molecules in the interstitial fluid. Cytotoxic edema is due to metabolic derangements within cells that lead to changes in osmolality, swelling, and death, often from inadequate metabolism or too severe ionic load for the membrane pumps to handle (reviewed in Greve and Zink, 2009; Zink et al., 2010; Villamar et al., 2012).

While the spinal cord shares certain mechanisms of injury with the brain, its injury patterns also have some important differences. For example, the most frequent primary cause of SCI is traumatic acute compression, and usually at least some subpial neurological tissue is preserved (Wolman, 1965; Tator and Edmonds, 1979; reviewed in Tator, 1995). Mechanical trauma preferentially affects the central gray matter of the cord, likely due to its vascularity and softer consistency, and can cause necrosis, edema, hemorrhage, and vasospasm (Wolman, 1965; reviewed in Tator, 1995). A cascade of secondary pathophysiology similar to that seen in the brain follows, including ischemia, apoptosis, necrosis, fluid and electrolyte disturbances, excitotoxicity, production of free radicals, lipid peroxidation, and an inflammatory response. These processes result in further neurological damage, swelling, and ischemia. Ultimately, a large fluid-filled cavity or cyst forms in the center of the injured cord surrounded by a subpial rim of preserved axons, many of which become demyelinated. Hypertrophic astrocytes and macrophages secrete extracellular matrix and inhibitory molecules that form a glial scar—a physical and chemical barrier to neural regeneration (reviewed in Tator and Fehlings, 1991; Tator, 1995; Mothe and Tator, 2012).

Although intuitively attractive, attempts to mitigate secondary injury and improve recovery with pharmaceutical therapies have been well studied with modest results at best (DeFina et al., 2010; reviewed in Breceda and Dromerick, 2013; Krieger, 2013). In the acute phase of brain injury, suppression of glutamatergic activity appears to be beneficial in minimizing neurological damage and disability (Liu et al., 2013). In the subacute phase, modulation of GABAergic inhibition can minimize the functional impact of an injury (reviewed in Demirtas-Tatlidede et al., 2012). Other drugs that have been used to enhance motor recovery after TBI and/or stroke include naltrexone, bromocriptine, fluoxetine, venlafaxine, levo/carbidopa, donepezil, modafinil, rivastigmine, desipramine, zolpidem, amantadine, methylphenidate, dextroamphetamine, and rasagiline (reviewed in DeFina et al., 2010; Breceda and Dromerick, 2013; Krieger, 2013). Most of these drugs are aimed at normalizing the electrochemical balance of the injured brain to optimize its ability to heal and minimize secondary injury. Dopaminergic medications have also been shown to promote gamma band activity during attention through D4 receptor activation (Kuznetsova and Deth, 2008). Nicotinamide too may help reverse severe oxidation, likely through mitochondrial mechanisms (Shetty et al., 2014), and some authors have suggested that blockade of AMPK in acute brain injury may protect against zinc neurotoxicity (Eom et al., 2016).

Selective serotonin reuptake inhibitors (SSRIs) like fluoxetine play a role in treating depressive symptoms that often accompany neurological disease (thought to be associated with disruption of corticostriatal and thalamocortical loops) (Terroni et al., 2011). Treating depression is crucial to recovery but is often overlooked, as the symptoms of depression and learned helplessness can be found in up to 30% of early stroke patients (Hackett et al., 2014). Vitamins and antioxidants such as essential amino acids, minerals, cofactors, and “immunonutrition” [omega 6 and omega 3 fatty acids, arginine, glutamine, ribonucleic acids (RNAs), mycelia extracts] have all demonstrated modest but generally benign results (DeFina et al., 2010). Additionally, recent evidence suggests that prophylactic anticonvulsants like phenytoin in stroke/TBI are associated with worse functional outcomes, possibly due to reduced axonal/growth cone bursting from sodium channel suppression which may inhibit rewiring (Bhullar et al., 2014). The optimal timing of seizure prophylaxis after brain injury, if beneficial at all, remains open to debate (Thompson et al., 2015).

## Molecular mechanisms of neural repair

In addition to the injury mechanisms and glial scar described above, there are many biological mediators that alter the ability of the CNS to repair itself after injury. Intrinsic factors including transcription factors (c-Jun, Atf3, Klf family, Stat3, Sox11, and Smad1) and regeneration-associated genes (Gap43, Cap23, Arg1, Sprr1a, Hspb1, MARCKS, stathmin family, SCG10 L1, P21/waf1, and tubulins) have been shown to alter restoration potential (Grenningloh et al., 2004; Carmichael et al., 2005; reviewed in Sun and He, 2010; Tedeschi, 2011). Phosphatase and tensin homolog (PTEN), a tumor suppressor, seems to play an important role as eliminating its gene has been shown to both prevent apoptosis and induce axon extension in injured retinal ganglion cells (RGCs) (Park et al., 2008; de Lima et al., 2012b). Deletion of Socs3, a suppressor of signaling through the Jak-STAT pathway, also promotes regeneration by enhancing the efficacy of ciliary neurotrophic factor (CNTF) (Smith et al., 2009). If the mechanistic target of rapamycin (mTOR) is inhibited, the regenerative effect of PTEN deficiency is eradicated, suggesting that axon regeneration induced by PTEN deletion is dependent on the mTOR pathway (Park et al., 2008). The proto-oncogene bcl-2 (and expression of its anti-apoptotic protein) also plays a key role in preventing cell death after injury, enabling axonal regrowth in RGCs with the presence of trophic factors and physiologic electrical activity (Chen et al., 1997; Goldberg et al., 2002).

**Figure 2:**
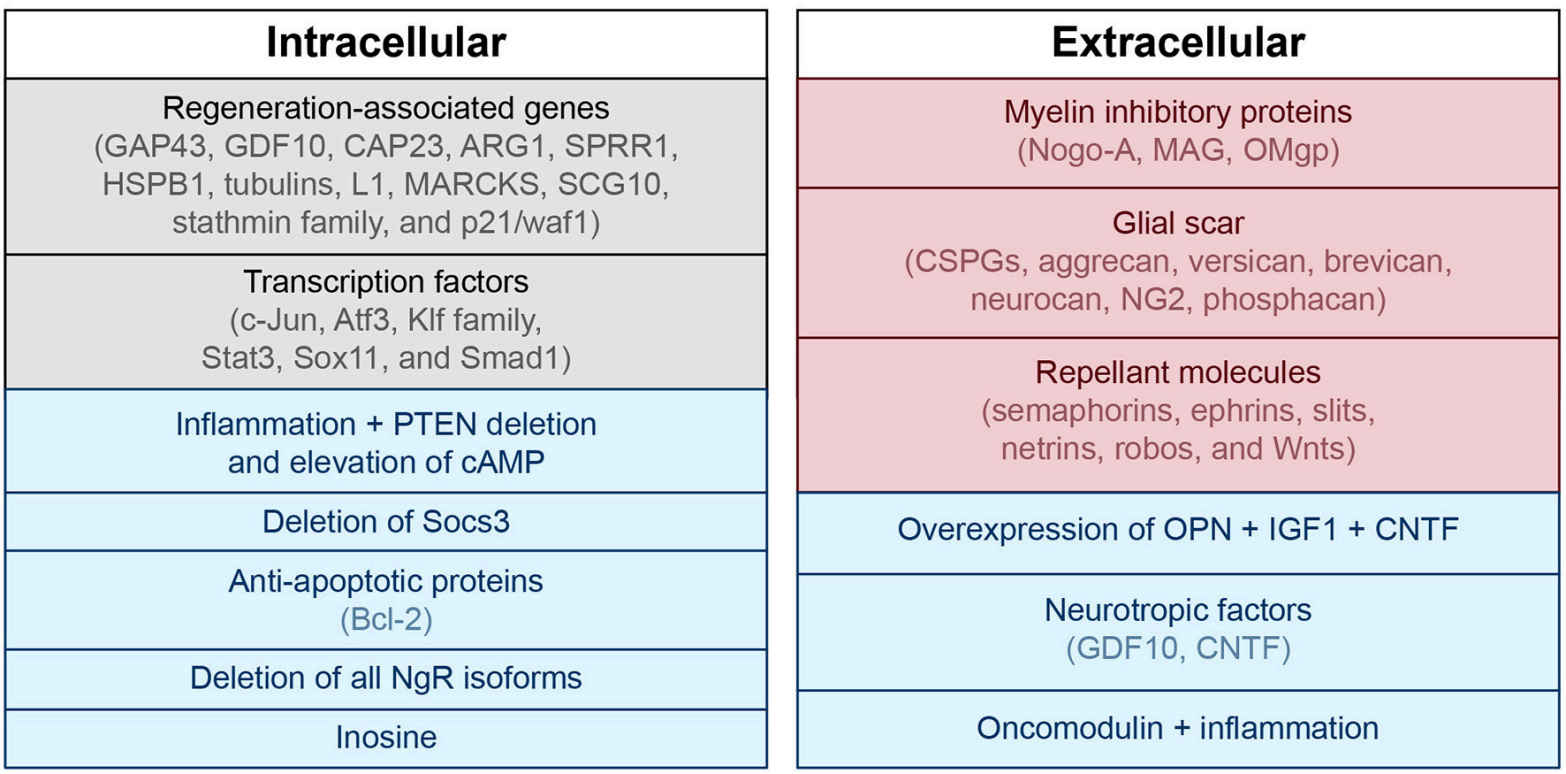
**Intra- and extracellular mechanisms of neuronal growth and inhibition**. Blue, associated with neuronal growth; Red, associated with neuronal inhibition; Black, modulates both neuronal growth and inhibition. PTEN, phosphatase and tensin homolog; cAMP, cyclic adenosine monophosphate; GAP43, growth associated protein 43; GDF10, growth differentiation factor 10; CAP23, cytoskeleton-associated protein; ARG1, arginase 1; SPRR1, small proline-rich protein 1; HSPB1, heat shock protein family B (small) member 1; MARCKS, myristoylated alanine-rich C-kinase substrate; SCG10, superior cervical ganglion 10; NgR, nogo receptor; CSPG, chondroitin sulfate proteoglycans; NG2, neural/glial antigen 2; MAG, myelin-associated glycoprotein; OMgp, oligodendrocyte-myelin glycoprotein; CNTF, ciliary neurotrophic factor; OPN, osteopontin; IGF1, insulin-like growth factor 1.

Extrinsic factors that prevent axonal regeneration include inhibitory proteins associated with myelin [e.g., NogoA, myelin-associated glycoprotein (MAG), and oligodendrocyte-myelin glycoprotein (OMgp)], proteoglycans in the perineuronal net and glial scar [e.g., chondroitin sulfate proteoglycans (CSPGs) like aggrecan, versican, brevican, neurocan, NG2, and phosphacan], and molecules that repel axon growth during development which continue to be expressed in the mature CNS (e.g., semaphorins, ephrins, slits, netrins, robos, and Wnts) (reviewed in Benowitz and Yin, 2007; Benowitz and Carmichael, 2010; de Lima et al., 2012a; Omura et al., 2015). A summary of intrinsic and extrinsic factors affecting neural growth and inhibition is provided in Figure 2.

Despite identification of these molecules as being important, removal or blockage of extracellular inhibitory factors alone so far has failed to achieve extensive axonal regeneration with a few exceptions (Alilain et al., 2011; reviewed in Benowitz and Yin, 2007; Benowitz and Carmichael, 2010; Liu et al., 2011). Interestingly, a strain of dorsal root ganglion neurons grown from CAST/Ei knockout mice are less inhibited by the same extrinsic cues listed above (Omura et al., 2015). Large regenerative responses have been noted in these cells, and activin seems to play an important role. Also, deletion of receptors that bind to myelin-associated inhibitory molecules (MAIs), or Nogo (NgR) receptors, has been shown to increase regeneration potential in neurons (Dickendesher et al., 2012). This is why anti-Nogo immunotherapies are currently of great interest as potential treatments for neurological injury (Lee et al., 2004; Freund et al., 2006; Maier et al., 2009; Wahl et al., 2014). In fact, immunotherapies aimed at blocking inhibitory factors like NogoA have successfully demonstrated increased sprouting associated with functional recovery in both rat (Bregman et al., 1995; Liebscher et al., 2005; Maier et al., 2009) and primate (Freund et al., 2006) models of SCI. In 2014, Wahl et al. published near full recovery of skilled forelimb function in rats with large strokes after intrathecal injection of an anti-NogoA antibody followed by intensive task-specific training (Wahl et al., 2014). Injection of the NogoA neutralizing agent was shown to promote growth of corticospinal fibers from the intact forebrain motor cortex across the midline of the cervical spinal cord to the hemicord that had lost its input from the motor cortex. This new fiber sprouting was then stabilized by a goal-directed physical therapy regimen. Interestingly, sequential application of drug then training was necessary to show benefit. When immunotherapy and forced-use training were combined simultaneously, functional outcome was *poorer* compared to no treatment at all or each treatment individually, likely due to abundant but aberrant fiber branching (also seen in Maier et al., 2009). This example outlines the important distinction between regrowth with synapse formation and the restoration of function.

Another example of this principle was demonstrated by Bei et al. in showing that PTEN/SOCS3 co-deletion or overexpression of osteopontin (OPN)/insulin-like growth factor 1 (IGF1)/CNTF could induce regrowth of adult mouse retinal axons to synapse in the superior colliculus, but this connection did not restore visual function on its own (Bei et al., 2016). In fact, these regenerated axons failed to conduct action potentials (APs) due to lack of myelination, and administration of voltage-gated potassium channel blockers was required to enable proper conduction and improve visual acuity.

Despite the litany of inhibitory mechanisms examined above, there are also signals released in the injured brain that are known to promote axonal growth after injury. For example, growth and differentiation factor 10 (GDF10) is induced in stroke and works through transforming growth factor beta receptors I and II (TGFβRI and TGFβRII) to promote axonal outgrowth (Li et al., 2015). Growth associated protein 43 (GAP43), a neuronal growth cone marker, is also induced in peri-infarct cortex after stroke (Stroemer et al., 1995; Schaechter et al., 2006). The purine nucleoside inosine works through a direct intracellular mechanism to induce expression of genes associated with axonal growth (e.g., GAP43, L1, and α-1 tubulin) and has been shown to induce axonal reorganization and improve behavioral outcomes after spinal cord injury and stroke (Zai et al., 2009, 2011; Kim et al., 2013), as well as restore levels of GAP43 in the hippocampus in rats after stroke (Chen et al., 2002; de Lima et al., 2012b; Dachir et al., 2014).

The role of inflammation in axonal regeneration is somewhat controversial. Some components of inflammation cause tissue damage and neuronal death (see The Biology of Neurological Injury section), while others promote cell survival, axon sprouting, and regeneration (Shetty and Turner, 1995; Yin et al., 2003; de Lima et al., 2012a; Kurimoto et al., 2013; Baldwin et al., 2015; reviewed in Benowitz and Popovich, 2011). Both oncomodulin, a macrophage-derived growth factor for RGCs, and injury-induced cytokine release appear to play a role in inflammation-induced axonal regeneration (Yin et al., 2006, 2009; Kurimoto et al., 2013). Traditional anti-inflammatory therapies (e.g., NSAIDs such as ibuprofen) may suppress beneficial as well as deleterious aspects of the immune response, and they can stimulate axonal regeneration via direct effects on neurons (reviewed in Benowitz and Yin, 2008; Benowitz and Carmichael, 2010; Benowitz and Popovich, 2011). When combined with PTEN deletion and elevation of cyclic adenosine monophosphate (cAMP), intraocular inflammation will enable some retinal ganglion cells to regenerate injured axons from the eye to the brain and restore simple visual responses (de Lima et al., 2012b).

## The physiology of recovery from neurological injury

In addition to the molecular and intracellular mechanisms mentioned above, proper function of the neuraxis also relies on the appropriate establishment and maintenance of intercellular mechanisms. Although the CNS does not fully self-repair after injury, neurogenesis does occur naturally in the healthy adult brain. This process happens primarily in the subgranular and subventricular zones (SGZ and SVZ, respectively), and it helps support learning, memory, and olfaction (Doetsch et al., 1999; Laywell et al., 2000; Seri et al., 2001; reviewed in Alvarez-Buylla and Lim, 2004; Ohab and Carmichael, 2008). These areas contain niches of progenitor, glial, and endothelial cells that can self-renew or differentiate into a glial or neuronal lineage. The SGZ supplies the dentate gyrus of the hippocampus and the SVZ gives cells to the olfactory bulb to integrate into local circuitry and support function. There is evidence that damage from stroke stimulates cell proliferation within these zones, and immature neurons are recruited into damaged areas of the striatum and cortex. This process starts at 2 weeks and lasts up to several months after injury (Macas et al., 2006; Thored et al., 2006). Initially, tens of thousands of immature neurons can migrate to damaged areas. However, few mature and survive long-term (Zhang et al., 2001; Arvidsson et al., 2002). While the generation and migration of new neurons to damaged areas is associated with functional recovery, it is possible that behavioral recovery is achieved through mechanisms other than neuronal replacement, e.g., growth factor production in local tissue (reviewed in Ohab and Carmichael, 2008).

Other more prominent mechanisms of restoration of function include reduction in edema, resolution of diaschisis (loss of function in connected areas of the brain due to inactivity, loss of blood flow, and decreased metabolism), and optimization of remaining motor areas (Nudo and Milliken, 1996; reviewed in Feeney and Baron, 1986). Neural plasticity and reorganization occur through the uncovering of previously latent synapses, collateral sprouting of synapses from nearby intact neurons, strengthening or weakening of existing synapses [e.g., through long-term potentiation (LTP) or long-term depression (LTD)], and changes in concentrations of neurotransmitters, ions, gap junctions, and glial cells (reviewed in DeFina et al., 2009; Demirtas-Tatlidede et al., 2012; Villamar et al., 2012; Nahmani and Turrigiano, 2014). These mechanisms are outlined in Table 2. While neural plasticity contributes significantly to functional recovery, it should be noted that not all types are beneficial. For example, maladaptive plasticity and inappropriate axonal sprouting can lead to spasticity, pathological pain, schizophrenia, and seizures (Dimitrijevi and Nathan, 1967; Flor et al., 1995; Teyler et al., 2001; Quartarone et al., 2008; Thickbroom and Mastaglia, 2009; Kuner, 2010; Hasan et al., 2011).

**Table 2:** **Mechanisms of neurological recovery**.

Reduction in edema	Long term potentiation or depression
Resolution of diaschisis	Local growth factor production
Optimization of remaining motor areas	Uncovering of latent synapses
Reorganization	Cellular proliferation/recruitment from SGZ/SVZ^*^
Collateral sprouting from local intact neurons	Long-range axonal sprouting (animal models)^*^
Changes in concentrations of neurotransmitters, ions, gap junctions, and glial cells	

* *Theoretical, or proven in animal models only. SGZ, subgranular zone; SVZ, subventricular zone*.

Though long-range axonal sprouting was once thought to be non-existent in adult mammals, evidence now supports this possibility in animal models (Chen et al., 2002; Liebscher et al., 2005; Freund et al., 2006; Maier et al., 2009; van den Brand et al., 2012; Wahl et al., 2014). Context-dependent cortical activity and functional growth cones paired with positive feedback seems to be critical for this type of axonal sprouting to generate robust and lasting functional improvement (van den Brand et al., 2012; Wahl et al., 2014). Even without long-range axonal sprouting, some level of functional improvement can occur via other mechanisms of neural plasticity. For example, after stroke, both hemispheres are known to assist with recovery depending on the size of the injury (Dancause, 2005; reviewed in Dancause and Nudo, 2011; Kantak et al., 2012). Following a small stroke within the primary motor cortex (M1) or the corticospinal tract, both ipsilesional dorsal and ventral premotor cortices (PMCs) can reorganize themselves. However, when a lesion involves a larger portion of M1 and the dorsal PMC, the contralesional PMC appears to be critical for recovery-related reorganization (reviewed in Dancause and Nudo, 2011; Kantak et al., 2012). Initiation of post-infarct axonal sprouting from the intact cortical hemisphere to peri-infarct cortex and the contralateral dorsal striatum is signaled by synchronous neuronal activity (Carmichael and Chesselet, 2002). In chronic stroke patients, activity in ipsilesional primary motor and medial-premotor cortices has been shown to be associated with good motor recovery, whereas increased cerebellar vermis activity signals poor recovery (Favre et al., 2014).

For up to several months after the initial injury, the neural environment remains conducive to recovery due to its relatively loose extracellular space, enhanced neurotrophic factors, open synaptic sites, and probing axonal growth cones (Napieralski et al., 1996; Carmichael et al., 2005; reviewed in Nudo, 2013). After 6 months to 1 year, injuries have been classically considered chronic with little opportunity for further gain, although this doctrine is beginning to change (Figure 1) (reviewed in Langhorne et al., 2011; Teasell et al., 2014).

## The neurophysiology underlying brain-machine and neural interface training

Neural- or brain-machine interfaces are electrode-computer constructs that extract and decode information from the nervous system to generate functional outputs. These have been developed to bypass motor lesions (assistive BMIs) (Wolpaw and McFarland, 1994; Kennedy and Bakay, 1998; Leuthardt et al., 2004; Moritz et al., 2008; Ethier et al., 2012; Collinger et al., 2013; Memberg et al., 2014; Jarosiewicz et al., 2015; Bouton et al., 2016; Capogrosso et al., 2016; Hotson et al., 2016; Rajangam et al., 2016; Vansteensel et al., 2016; reviewed in Lobel and Lee, 2014) and, more recently, to facilitate neural plasticity and motor learning to enhance recovery after injury (rehabilitative BMIs) (Carhart et al., 2004; Buch et al., 2008; van den Brand et al., 2012; Ang et al., 2013; Ramos-Murguialday et al., 2013; Wahl et al., 2014; Gharabaghi et al., 2014a,b,c; Gerasimenko et al., 2015b; Donati et al., 2016; reviewed in Ethier et al., 2015; Jackson and Zimmermann, 2012).

Interfaces generally contain at least four components: (1) a method of extracting signals from the nervous system, (2) a way to decode the signals to predict user intent, (3) an output to affect the subject's environment, and (4) a feedback system to help the user refine the output (e.g., visual or other sensory modality). Means of extracting nervous system signals range from invasive [intracortical microelectrodes (APs, or spikes) and larger scale sub- or epidural electrodes (electrocorticography, ECoG)] to non-invasive [electroencephalography (EEG) or electromyography (EMG)]. Targeted outputs have included cursors on a screen (Wolpaw and McFarland, 1994; Kennedy and Bakay, 1998; Leuthardt et al., 2004; McFarland et al., 2010), virtual typing (Jarosiewicz et al., 2015), robotic or prosthetic arms (Collinger et al., 2013; Hotson et al., 2016), wheelchairs (Rajangam et al., 2016), exoskeletons (Donati et al., 2016), the spinal cord (Zimmermann and Jackson, 2014; Capogrosso et al., 2016), and a patient's own extremities (Ethier et al., 2012; Memberg et al., 2014; King et al., 2015; Bouton et al., 2016; Vidaurre et al., 2016).

While initially developed due to the belief that the human nervous system could not self-regenerate (as examined in the previous sections), BMIs have also led to exciting and innovative ways to understand and interact with the nervous system. Current understanding of brain function recognizes an intricate arrangement of interconnected units and circuits that contribute to a larger performing network, as opposed to older models which viewed the brain as a collection of independent anatomical modules with discrete functions (Breakspear and Stam, 2005; Serences and Yantis, 2006; reviewed in Meunier et al., 2010). It has been shown that functionally coupled remote brain locations display near synchronous discharges that represent emergent properties of their assimilated networks (Breakspear and Stam, 2005; Womelsdorf et al., 2007; Stevenson et al., 2012; Menzer et al., 2014). This dispersion of information likely explains why motor information can be found widely distributed throughout the cortex, and how random samples of neurons can provide enough information to reconstruct certain movements in great detail (Carmena et al., 2003; Fitzsimmons et al., 2009; reviewed in Nicolelis and Lebedev, 2009). However, it has also been shown that no matter how well tuned a single neuron is to a behavioral task, that an individual cell only contains limited information and can vary greatly over a short period of time (Wessberg et al., 2000; Carmena et al., 2003). Interestingly, once an ensemble of neurons reaches a certain size, its collective predictive ability plateaus, suggesting that there is redundancy in the neuronal network, and that there are a critical number of neurons required to decode motor intention (Carmena et al., 2003; Vargas-Irwin et al., 2010; reviewed in Donoghue, 2008; Nicolelis and Lebedev, 2009). Collectively, these concepts have led to population algorithms, or decoders that exploit the idea that individual neurons encode multiple parameters with different weights and may vary from trial to trial; however, useful information is maintained among a population instead of individual neurons. The advantage of population decoding systems is that they work even when individual neurons poorly encode motor behavior. These algorithms, in addition to advances in technology that have enabled large-scale recordings of single-neuron activity patterns, have led to the success of many BMIs described in the following section.

The true language of the motor cortex, or how the motor cortex encodes its output signals, is a subject of debate (Vargas-Irwin et al., 2010; Cherian et al., 2011). Coordinated actions of the limbs may engage widespread cortical areas, and M1 is known as the site where motor plans tend to merge before diverging to multiple muscle groups (Vargas-Irwin et al., 2010). While M1 clearly contains kinematic information (joint position and trajectory) sufficient for accurate predictions (Vargas-Irwin et al., 2010), there is evidence that it may more directly encode kinetic (force) variables (Morrow et al., 2007; Cherian et al., 2011; Flint et al., 2014). This would suggest that BMIs built to encode force and/or EMG signals may be more robust across different positional dynamics than trajectory-based BMIs. For gait decoding, there is evidence that motor cortex BMIs may perform better when estimating gait phases or locomotor behaviors as opposed to continuous kinematic variables of leg movement (Rigosa et al., 2015). Interestingly, bimanual arm control appears to have its own representation in the cortex and does not seem to be described simply by a superposition of unilateral movements (Ifft et al., 2013).

**Figure 3:**
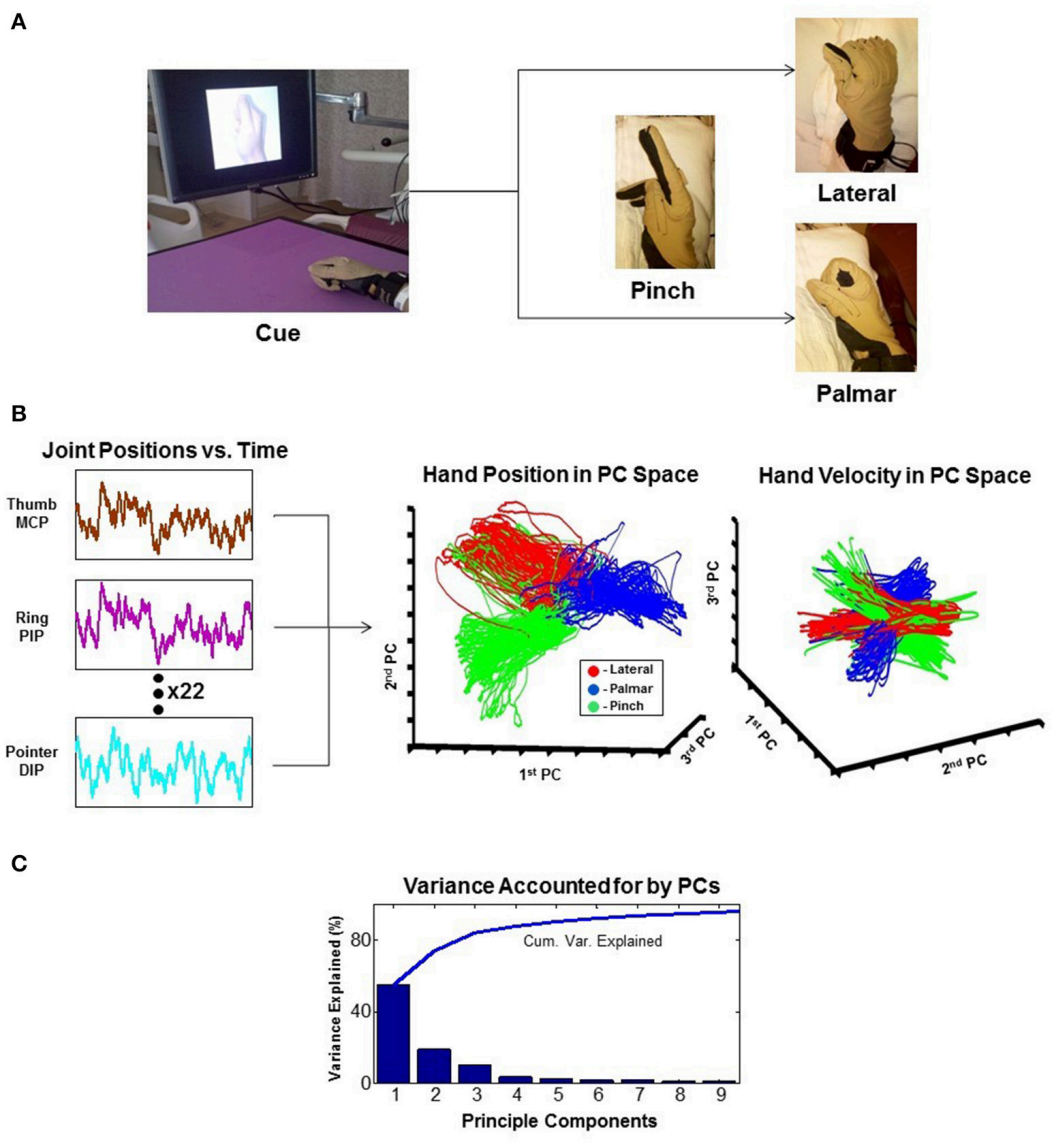
**Principal component analysis (PCA)**. The example reduces 22 joint-position variables of the wrist and fingers to 3 dimensions that represent the state of the hand. **(A)** Experimental task where the patient is cued to move his hand into one of the three configurations shown. **(B)** 22 independent variables of hand movement are recorded and reduced to 3 principle components (PCs). Results are plotted as hand position and hand velocity in 3-dimensional PC space. **(C)** Greater than 80% variance of hand movement is accounted for using only the first three PCs (Krucoff and Slutzky, 2011). MCP, metacarpophalangeal joint; PIP, proximal interphalangeal joint; DIP, distal interphalangeal joint.

As attempts for more dexterous and intuitive control of neural interfaces are pursued, the question of how many independent control signals can be extracted from a neural ensemble arises. The human arm has 7 degrees of freedom (DOF), and the hand has more than 20 (Stockwell, 1981; Jones, 1997). As it turns out, natural grasp postures and reaching-to-grasp movements exist in a much smaller subspace than physically possible movements (Ingram et al., 2008). Dimensionality reduction techniques like principal component analysis (PCA) show that a large proportion of the variance of natural grasping behaviors can be represented by two to three principal components. This provides a strategy for neural interfaces to recapitulate potentially more complex appearing movements while extracting fewer degrees of freedom (reviewed in Hatsopoulos and Donoghue, 2009). An example of such a technique is shown in Figure 3 (Krucoff and Slutzky, 2011).

**Figure 4:**
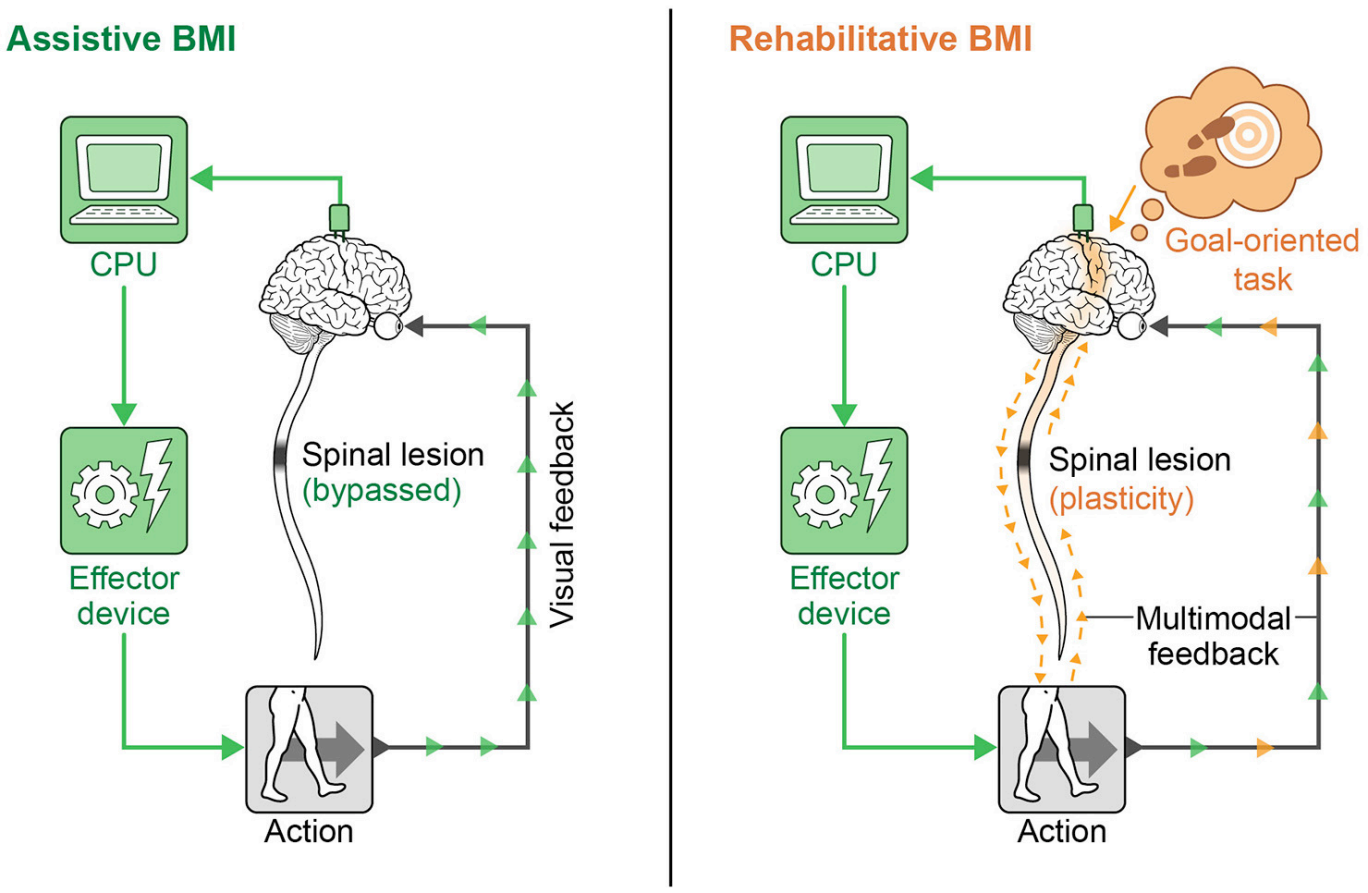
**Assistive vs. rehabilitative BMIs**. The assistive BMI uses brain signals to bypass a neural lesion and generate an intended action. The rehabilitative BMI pairs goal-oriented tasks with positive feedback and works to re-activate lesioned circuits to create plasticity for long-lasting functional improvement.

In recent years, neural interfaces have been developed to modulate neural plasticity and enhance recovery (rehabilitative BMIs) in addition to bypassing lesions (assistive BMIs). The transition from assistive to rehabilitative BMIs has come with the realization that patients with a chronic neurologic injury may not be at a static level of functioning as previously thought, and that underlying networks even in chronic injury can be modified over time (Guggenmos et al., 2013; reviewed in Jackson and Zimmermann, 2012). A diagram showing the conceptual difference in approach to assistive vs. rehabilitative BMIs is provided in Figure 4. Rehabilitative BMIs pair goal-oriented tasks with expected outcomes and work to activate lesioned circuits to create plasticity for long-lasting improvements. This approach takes advantage of a principle called spike timing dependent plasticity (STDP), or Hebbian plasticity (often expressed as, “neurons that fire together wire together”) (Hebb, 1949; Cooper, 2005). This is the idea that synaptic strength is redistributed to favor functionally relevant pathways that are coincidently active, inferring that both the sign and magnitude of synaptic modification are determined by the precise timing of APs (Rebesco and Miller, 2011). The best known example of Hebbian plasticity is perhaps LTP and LTD in memory circuits. For modeling of complex, larger scale circuits, the Bienenstock–Cooper–Munro (BCM) model maybe more representative of behavior. This theory incorporates both presynaptic and postsynaptic firing rates (Bienenstock et al., 1982; reviewed in Cooper and Bear, 2012) and applies a sliding threshold for LTP/LTD based on post-synaptic activity as the metric for stabilization.

A related concept employed in some rehabilitative BMIs is paired associative stimulation (PAS), or the act of pairing stimulation sites to promote plasticity (Stefan et al., 2000; Carson and Kennedy, 2013). An example of a commonly used central stimulation strategy is transcranial magnetic stimulation (TMS). TMS involves applying rapidly changing magnetic fields to the scalp via a magnetic stimulator. Continuous low frequency repetitive stimuli (≤1 Hz rTMS) decreases excitability of targets areas (similar to LTD which is maximally evoked at 1 Hz), while bursts of intermittent high frequency stimuli (≥5 Hz rTMS) enhance excitability (similar to LTP with high frequency bursts) (Kobayashi and Pascual-Leone, 2003; Demirtas-Tatlidede et al., 2012; Shin et al., 2014). These techniques have been used to induce modulation across cortico-subcortical and cortico-cortical networks through trans-synaptic spread, resulting in distant but specific changes along functional networks. Evidence suggests long term effects from TMS may be related to modulation of NMDA glutamatergic receptors, similar to induction of LTP/LTD (reviewed in Villamar et al., 2012). If timed correctly, corresponding sensory inputs can be potentiated (Stefan et al., 2000). Functional electrical stimulation (FES) of paralyzed muscles or electrical stimulation of the nervous system distal to the injury timed with voluntary effort has been shown to accelerate recovery in both SCI and stroke (Daly et al., 2006; Jung et al., 2009; Popovic et al., 2011; Kafri and Laufer, 2015).

## The state of the art in neural prostheses and brain-machine interfaces

The first assistive BMI for patients with severe motor impairment was developed for patients with locked-in syndrome—a condition where a patient is cognitively aware of his or her environment but is unable to move or make sounds (Kennedy and Bakay, 1998; Birbaumer et al., 1999). Birbaumer et al.'s device was an EEG-based system designed to translate purposeful slow cortical potentials (SCPs) into a binary selection of letters or words on a screen. Since then, EEG-based systems have advanced to move cursors on a screen in up to 3 dimensions (McFarland et al., 2010), open and close a hand orthosis (Ramos-Murguialday et al., 2012), provide limited FES-control of upper and lower extremities (King et al., 2015; Vidaurre et al., 2016), and ambulate a lower extremity exoskeleton (Donati et al., 2016); however, their potential is hindered due to inherently poor reliability, latency, signal variability, and generally non-intuitive nature (reviewed in Jackson and Zimmermann, 2012).

**Figure 5:**
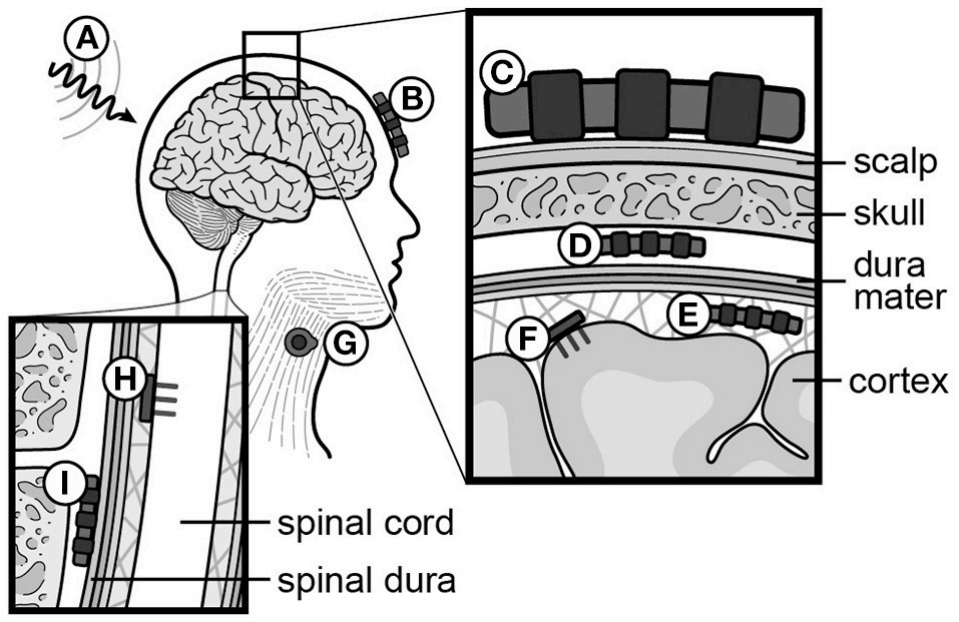
**Recording and stimulating modalities for neural signals**. **(A)** Functional MRI (fMRI), **(B)** functional Near-Infrared Spectroscopy (fNIRS), **(C)** scalp electrodes (EEG), **(D)** epidural electrodes (FP), **(E)** subdural electrodes (ECoG), **(F)** intracortical electrodes (AP, or spikes), **(G)** muscle electrodes (EMG), **(H)** intraspinal electrodes (AP, or spikes), **(I)** spinal epidural electrodes (FP). EEG, electroencephalography; FP, field potential; ECoG, electrocorticography; AP, action potential; EMG, electromyography.

In non-invasive BMIs, six types of brain signals have been tested: sensori-motor rhythms (SMRs, 8–15 Hz, i.e., rolandic alpha or mu rhythm) (Pfurtscheller et al., 1992, 2006; Wolpaw and McFarland, 1994; McFarland et al., 2006), event-related potentials (ERPs) (Farwell and Donchin, 1988), SCPs (Birbaumer et al., 1999), steady-state visually or auditory evoked potentials (SSVEPs/SSAEPs) (Sakurada et al., 2013), concentration changes of oxy/deoxy hemoglobin using functional near-infrared spectroscopy (fNIRS) (Sitaram et al., 2009; Mihara et al., 2013; Rea et al., 2014), and blood-oxygenation level dependent (BOLD)-contrast imaging using functional MRI (Weiskopf et al., 2003). Implantable BMIs utilize epidural, subdural, or intracortical electrodes. Epi- and subdural arrays record field potentials (FPs), while intracortical electrodes record APs (a.k.a. spikes) (reviewed in Soekadar et al., 2015). A summary of neural recording methods is provided in Figure 5.

Of all recording modalities, intracortical systems have demonstrated the most advanced control potential. Hochberg et al. provided initial evidence that a chronically tetraplegic human could operate a BMI using electrodes implanted into the arm area of M1 to drive a computer cursor on a screen (Hochberg et al., 2006). Several years later, that same group demonstrated brain control of a robotic arm to perform three-dimensional reach and grasp movements in two patients, including the ability to drink coffee from a bottle (Hochberg et al., 2012). Since then, intracortical BMIs have enabled brain-controlled typing (Jarosiewicz et al., 2015; Nuyujukian et al., 2016), driven seven degrees of freedom in a prosthetic limb at over 90% accuracy (Collinger et al., 2013), restored voluntary movement and grasp via real-time FES to a monkey with a paralyzed hand (Moritz et al., 2008; Ethier et al., 2012), coordinated cervical intraspinal simulation to enact reach and grasp in the upper extremities (Zimmermann and Jackson, 2014), restored functional hand movement to a patient with quadriplegia (Bouton et al., 2016), and alleviated gait deficits in macaques with hemicord injuries via brain-controlled spinal epidural stimulation (Capogrosso et al., 2016). Additionally, intracortical electrodes distributed in more diffuse frontoparietal areas have enabled simultaneous bimanual control of avatar arms (Ifft et al., 2013).

To date, the only recording modalities stable enough to drive assistive BMIs over a prolonged period of time are implanted electrodes. Even here, the major concern with chronically implanted intracortical sensors is signal longevity. Microelectrode arrays (MEAs) face gliosis that begins in the first few months and can lead to failure at an average of 1 year (Barrese et al., 2013, 2016). Eventually, progressive meningeal fibrosis can encapsulate and elevate a microarray out of the cortex. Foreign body reactions and reactive oxygen species lead to degradation of the materials over time. In the most commonly used MEA known as the Utah array (UEA), failure can be due to cracking of parylene, corrosion of platinum, and delamination of silicone elastomer (Barrese et al., 2013, 2016). Other intracortical recording techniques have been developed in non-human primate models that minimize cortical damage and have recorded stable signals for years, but these systems are not widely adopted and have not yet been demonstrated in human patients (Krüger et al., 2010; Schwarz et al., 2014).

This concern for longevity is one of the reasons ECoG-based systems have become increasingly popular, as there is evidence that FPs may have greater longevity and stability than spikes (Flint et al., 2013, 2016). Recently, a fully implantable ECoG system has been used to create a typing interface for a locked-in patient with ALS by decoding hand motor intention (Vansteensel et al., 2016), and another 64 channel completely implantable system has been CE marked in Europe for human use in BCI applications (Mestais et al., 2015). FPs recorded from the surface of the human motor cortex contain high gamma activity (70–300 Hz) and time domain features that can be used to decode continuous force, isometric pinch force, and muscle activity in finger flexors with high levels of accuracy (Crone et al., 2001; Flint et al., 2014). Hotson et al. recently utilized a high-density ECoG array over the motor cortex to achieve control of individual fingers on a prosthetic limb by a human subject with epilepsy (Hotson et al., 2016). ECoG-based systems have also been used as a mechanism to provide therapy to chronic stroke patients who would otherwise be unable to participate (Buch et al., 2008; Ramos-Murguialday et al., 2013; Gharabaghi et al., 2014b). In one example, a patient gained volitional control of a feedback device and engaged himself in repetitive, high-intensity exercises of finger pointing and wrist extension without the need for a therapist. He could simultaneously monitor his own ability to modulate his brain activity and receive immediate rewards for success, eventually improving the function of the targeted muscle (Gharabaghi et al., 2014c). Additionally, there is now evidence that epidural arrays can provide similar information to subdural arrays regarding finger kinematics, thus potentially providing a less invasive and equally viable option for such applications (Flint et al., 2017).

Other implanted devices include cortex-to-cortex BMIs that have been used to bridge damaged neural pathways directly. Guggenmos et al. showed that a neural prosthetic could help reconnect premotor to somatosensory cortex in an injured rat brain to restore reach and grasp functions to pre-lesion levels (Guggenmos et al., 2013). Other investigators have connected the brains of several animals into a single system, or “brainet,” where the animals try to achieve a single objective using brain control only (Pais-Vieira et al., 2015; Ramakrishnan et al., 2015). Clinical applications for the latter remain to be established.

Novel methods for extracting brain signals are continually being developed. For example, Oxley et al. recently deployed an endovascular venous electrode array, or “stentrode,” into a superficial cortical vein over the motor cortex in freely moving sheep for over 6 months. They report recording performance similar to epidural surface electrodes (Oxley et al., 2016). Clinical applicability of this device remains to be seen, however, and its use will likely be limited to recording from cortical areas near its deployment. Furthermore, implantation of the electrodes will contain the risks associated with angiographic deployment of a stent, including hemorrhage, thrombosis, stroke, and infection. Notably, many of these limitations and risks are shared with other implantable electrode systems.

Despite the success of cortically controlled motor prosthetic devices in animals, the translation into broad clinical applicability for human patients remains a challenge (reviewed in Patil and Turner, 2008; Turner et al., 2008; Nicolelis and Lebedev, 2009). Although rarely studied in the context of a motor BMI, subcortical regions such as the motor thalamus and subthalamic nucleus (STN) are also involved in motor planning and execution (Friehs et al., 2004; Patil et al., 2004; Hanson et al., 2012; Ryapolova-Webb et al., 2014). Deep brain stimulation (DBS) in these areas is approved by the United States Food and Drug Administration (FDA) for the treatment of Parkinson Disease and tremor, and it is generally well-tolerated. For these reasons, some researchers have suggested that chronic subcortical ensemble recordings may enhance the viability of subcortical BMI systems (Hanson et al., 2012; Ryapolova-Webb et al., 2014). Recordings from microwire arrays in awake patients undergoing deep brain stimulation found that 61% of neurons in the STN and 81% of ventralis oralis posterior (VOP) and ventralis intermedius (VIM) neurons modulate with gripping force, and that ensembles of 3–55 simultaneously recorded neurons contained sufficient information to predict gripping force (Patil et al., 2004). In other studies, after minutes of practice, patients have been able to bring a cursor to a target (Friehs et al., 2004). Additionally, modulations in firing rate of neurons in VOP, VIM, and STN have been shown represent target onset, movement onset/direction, and hand tremor (Hanson et al., 2012).

In addition to brain signals, muscles proximal to an injury have been used as command signals in FES systems. For example, EMGs from muscles in the head and neck (e.g., platysma, trapezius, and auricularis) have been used to drive FES systems in patients with high cervical spinal cord injuries (Memberg et al., 2014). While these patients achieved enough voluntary control of their upper extremities to help with some activities of daily living (ADLs), their extremities did not reach antigravity strength, and there were serious limitations due to limb spasticity. Notably, EMG-based FES systems also do not work on patients who cannot voluntarily contract any muscles, and they require the use of pre-programmed stimulation patterns which can be non-intuitive (Memberg et al., 2014). Another major challenge facing BMIs on their way to useful clinical application is the difficulty in providing sensory or non-visual regulatory feedback to the user. While several groups have provided some level of information to a subject by stimulating the somatosensory cortex (London et al., 2008; Fagg et al., 2009; O'Doherty et al., 2009; Flesher et al., 2016), this technology remains in its infancy and is ripe for breakthrough. Improving artificial somatosensory feedback through intracortical microstimulation (ICMS), intraspinal stimulation, epidural spinal stimulation, or optogenetic stimulation are all efforts currently being undertaken (reviewed in Lebedev et al., 2011). However, perhaps the biggest hindrance to the large-scale development of BMIs is the lack of commercial appeal and marketability. Research and development costs of BMIs are enormous, and the market tends to be very niche and individualized. Until there is a large scale commercial interest, the greatest utility in advancing this field maybe in better understanding how to manipulate and rehabilitate the nervous system. While the cost of capable processors and electronics lessens with time, the cost of clinical trials remains a huge barrier to progress. Whether technological advancement, commercial interest, population demand, and clinical trial expense will evolve to allow for wide-scale testing and implementation remains to be seen (Patil and Turner, 2008). Also, for clinical viability, any interface needs to be reliable for a very long period of time (e.g., a decade), and, unfortunately, this remains a problem for virtually every studied neural implant except DBS electrodes. Future microelectrode arrays could address concerns about durability through improved insulation materials, inert electrode alloys, and/or incorporation of anti-inflammatory material along the lines of drug eluting stents (Barrese et al., 2016).

While largely outside the scope of this paper, interfaces for applications beyond motor recovery are also emerging. Devices designed to control medically refractory seizures are on the market. Neuromodulation for psychiatric illness is also currently being tested in many centers around the world, although results to date are mixed (Houeto et al., 2005; Mayberg et al., 2005; Greenberg et al., 2006). New targets for neuromodulation, including the fornix and septal area (Sweet et al., 2014), are under investigation for the treatment of Alzheimer's disease (Laxton et al., 2010). One can envision the development of neural interfaces for neuroprotection, memory (Hamani et al., 2008; Suthana et al., 2012), cognitive enhancement, concussion treatment, and sensory augmentation in the near future (reviewed in Laxton et al., 2013; Bick and Eskandar, 2016).

## Neurorehabilitation and electrical stimulation of the nervous system

Given that injured cells of the CNS do not regenerate on their own, how can recovery be facilitated for patients with TBI, stroke, or SCI? Several avenues for therapy have been explored, including various physical rehabilitation paradigms (Wernig and Müller, 1992; Protas et al., 2001; Taub and Morris, 2001; Taub et al., 2002; Duncan et al., 2011; Harkema et al., 2012; Mackay-Lyons et al., 2013) and electrical stimulation modalities (Shik and Orlovsky, 1976; Dimitrijevic et al., 1998; Rattay et al., 2000; Carhart et al., 2004; Minassian et al., 2004, 2007; Levy et al., 2008; DeFina et al., 2010; Dy et al., 2010; Troyk et al., 2012; Gad et al., 2013a,b; Angeli et al., 2014; Gerasimenko et al., 2015a,b; Prochazka, 2016). Individually each of these areas has yielded only modest results; however, by combining and improving techniques, significant progress has been made (Carhart et al., 2004; reviewed in Breceda and Dromerick, 2013).

**Figure 6:**
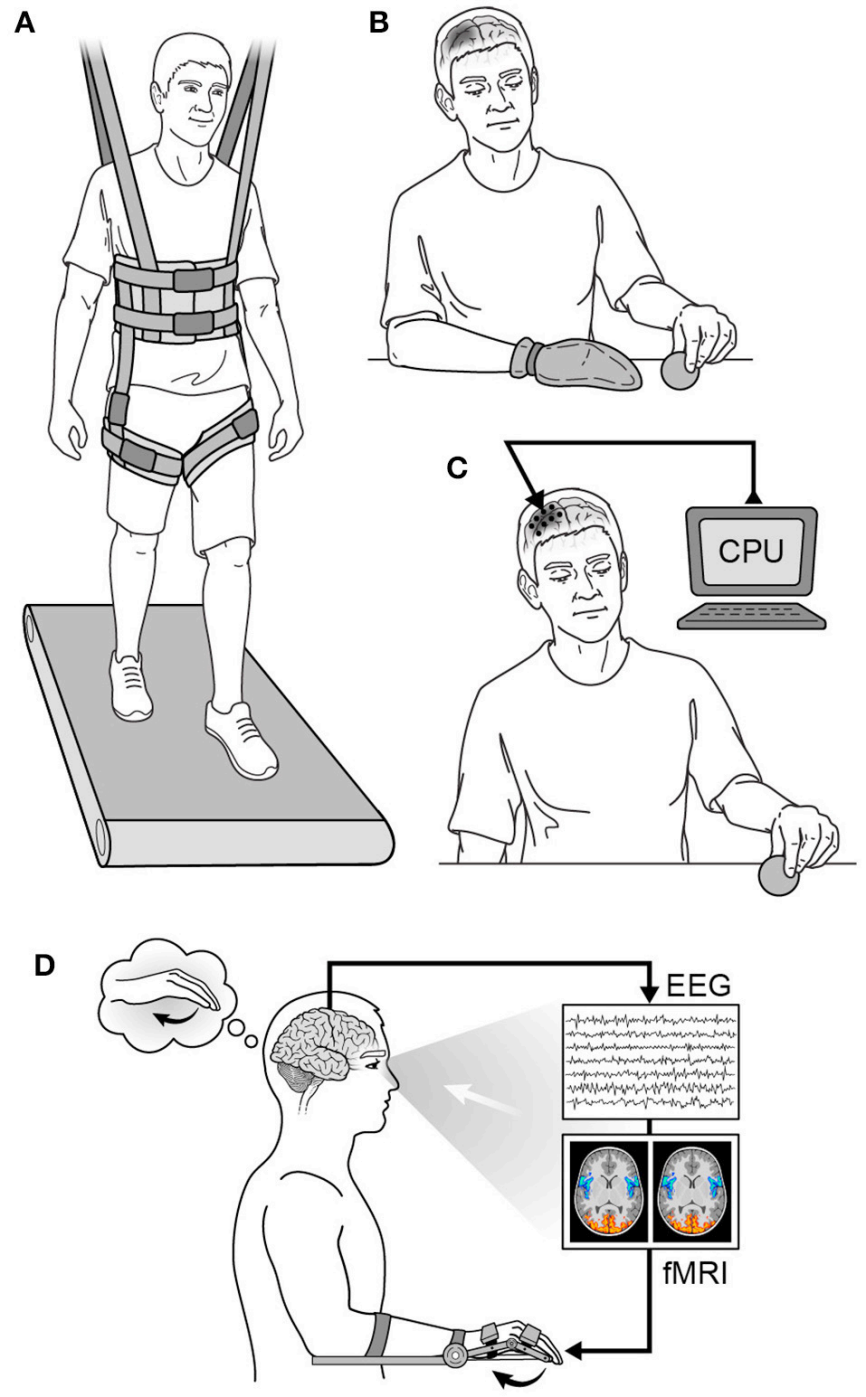
**Neurorehabilitation strategies. (A)** Partial weight-based therapy (PWBT), **(B)** constraint-induced movement therapy (CIMT) in a patient with a right hemispheric injury, **(C)** cortical neurostimulation in a patient with a right hemispheric injury, **(D)** biofeedback in a patient with a left hemispheric injury. CPU, computer processing unit; EEG, electroencephalography; fMRI, functional magnetic resonance imaging.

Physical therapy is the mainstay of virtually every neurorehabilitation program, and there have been several types of physical therapy studied. Examples of prominent physical therapy modalities are shown in Figure 6. For early stroke patients, constraint-induced movement therapy (CIMT) has been used to encourage use of the paretic limb by restraining the less affected one (Taub and Morris, 2001; Taub et al., 2002). This is done to avoid “learned non-use,” as animal data has shown maladaptive changes and worse functional outcomes from allowing overcompensation with the less affected limb to dominate goal-directed tasks (Allred et al., 2005; Allred and Jones, 2008). Timing the application of CIMT appears to be crucial, however, as behavioral interventions employed too early after injury may be deleterious (Kozlowski et al., 1996). The mechanism of worse outcome in too-early therapy is likely related to glutamate-NMDA excitotoxicity due to over-engaging vulnerable tissue surrounding the injury site (see The Biology of Neurological Injury section above). Additionally, early intensive training with immunotherapy has been shown to induce hyperinnervation and aberrant growth, resulting in wrong circuit connectivity and impaired function in rat models of stroke (see Molecular Mechanisms of Neural Repair section above) (Wahl et al., 2014). Several studies have examined the efficacy of CIMT for motor recovery in human stroke patients with mixed results (Wolf et al., 2006; Dromerick et al., 2009; McIntyre et al., 2012), and the optimal timing for its application in human stroke patients is yet to be determined (reviewed in Lang et al., 2015).

Another commonly used physical therapy paradigm is partial weight-bearing therapy (PWBT), or body weight supported treadmill training (BWSTT) (Wernig and Müller, 1992; Protas et al., 2001; Duncan et al., 2011; Mackay-Lyons et al., 2013). This model has evolved from observations that spinalized animals on a moving treadmill can initiate and sustain full weight-bearing stepping over different speeds and directions due to intrinsic changes in the locomotive rhythm generators of the spinal cord (Barbeau and Rossignol, 1987; reviewed in Wolpaw and Tennissen, 2001). When applied as a monotherapy to human stroke patients, benefits are largely comparable to those seen with other methods of physical therapy—the main value being increased cardiovascular endurance (Duncan et al., 2011; Mackay-Lyons et al., 2013). However, when used in patients with incomplete SCI either alone or in combination with FES and monoaminergic excitation, PWBT/BWSTT has been shown to facilitate locomotor function and decrease the reliance on assistive devices while improving coordination, speed, and endurance (Wernig and Müller, 1992; Protas et al., 2001; Harkema et al., 2012). In some cases, the benefits have not been limited to treadmill ambulation, but have been shown to be transferable to over-ground walking (Wernig et al., 1998; Carhart et al., 2004). In at least one patient with chronic incomplete SCI, intermittent PWBT alone was not sufficient to improve ambulation over ground; however, with the addition of epidural spinal stimulation, he was successful (Carhart et al., 2004).

Epidural spinal stimulation is one example of an emerging spinal modulation strategy to capture dormant but functional inter-neuronal pools distal to a spinal lesion and produce coordinated limb activity (Iwahara et al., 1992; Dimitrijevic et al., 1998; Sayenko et al., 2014; reviewed in Edgerton et al., 2004; Fong et al., 2009; Roy et al., 2012). Spinal stimulation to enhance motor performance in human patients with upper motor neuron disease (e.g., multiple sclerosis) was demonstrated as early as 1973 (Cook and Weinstein, 1973; Illis et al., 1976; Dooley and Sharkey, 1977–1978; Dimitrijevic et al., 1980, 2015), followed later by evidence that central pattern generators exist within the mammalian lumbosacral spinal cord that could be stimulated to produce locomotion (Grillner, 1985; Iwahara et al., 1992; Dimitrijevic et al., 1998; Minassian et al., 2007). Using electrical techniques to generate stepping in combination with extensive goal-directed physical therapy, patients with chronic and complete SCI have now shown the ability to develop positive functional plasticity and regain some voluntary control of lower extremity movement (Gad et al., 2015; Gerasimenko et al., 2015a,b). Other subjects with complete SCIs have regained the ability to selectively move their hips, knees, and ankles, as well as regain some coordination of flexor and extensor muscles (Sayenko et al., 2014; Dimitrijevic et al., 2015). Furthermore, some of these patients learned how to stand independently and activate lower limb musculature during partial weight-bearing stepping (Sayenko et al., 2014). In addition to attaining better lower extremity control, improvements in cardiovascular, temperature, bladder, and bowel control have been noted, as well as enhanced sexual function in some (Harkema et al., 2011; Angeli et al., 2014; Gad et al., 2015; Gerasimenko et al., 2015a,b). In other subjects, subthreshold epidural stimulation between L2 and S1, or direct stimulation of the pudendal nerve, has been shown to initiate micturition (Gad et al., 2014; McGee and Grill, 2014; McGee et al., 2015).

The mechanism by which complete and chronic SCI patients regain voluntary motor control of their lower extremities is currently unclear (reviewed in Kern et al., 2005). The fact that positive, lasting plasticity (up to years after training) can be induced suggests that there are likely subclinical surviving descending tracts in the injured spinal cord that are amenable to modulation and strengthening (Sherwood et al., 1992; Harkema et al., 2011; Angeli et al., 2014; Gerasimenko et al., 2015b). However, Van den Brand et al. showed that pairing goal-directed training with electrochemically enabled lumbosacral neurons (epidural stimulation with pharmacologic enhancement) can induce growth of *de novo* brainstem and intraspinal relays that re-enable voluntary control of locomotion in rats (van den Brand et al., 2012). As a control, they showed that automated treadmill-restricted training, which did not engage cortical neurons, failed to promote plasticity across the lesion or functional recovery. These results suggest that active, goal-directed training that engages task-specific cortical neurons is an essential component to recovery, and that its pairing with appropriate sensory cues (in this case, stepping gate) can lead to trans-lesional axonal sprouting and regained voluntary control of function (van den Brand et al., 2012). Interestingly, there is also evidence that spinal cord stimulation can replenish progenitor cells in the injured spinal cords of rat models (Becker et al., 2010), but this has not yet been explored in higher animals.

When used for SCI rehabilitation, epidural stimulation is often combined with monoamine therapy to increase the baseline excitatory state of distal functional elements and prime them for activation (Carhart et al., 2004; Harkema et al., 2011; van den Brand et al., 2012; Angeli et al., 2014; Wahl et al., 2014; Gerasimenko et al., 2015a,b). Neurons are known to release serotonin (5HT), norepinephrine (NE), and dopamine (DA) during locomotion within most laminae of lumbosacral segments (reviewed in van den Brand et al., 2015). These monoamines are thought to operate through volume neurotransmission (i.e., peri-synaptic signal diffusion), and, along with epidural stimulation, have helped promote locomotion in both animal models and human patients with incomplete and complete spinal cord injuries (Carhart et al., 2004; Harkema et al., 2011; van den Brand et al., 2012; Angeli et al., 2014; Wahl et al., 2014; Gerasimenko et al., 2015a,b).

More intricate and invasive methods of stimulating and recording from the spinal cord are also being developed (Troyk et al., 2012; Gad et al., 2013a; Chang et al., 2014; Grahn et al., 2015; Minev et al., 2015; Mazurek et al., 2016; Prochazka, 2016). New generations of intraspinal microstimulator systems, including wireless ones, are being constructed to take advantage of this residual functional potential and plasticity. Older intraspinal stimulation technology was designed primarily for pain suppression, so there is optimism that these newer and more targeted devises will generate better outcomes than previously seen.

Direct brain stimulation as an adjunct to physical therapy has shown the ability to enhance functional recovery of reach and grasp tasks in rats to pre-lesion levels (Kleim et al., 2003; Guggenmos et al., 2013) and enhance cortical plasticity and functional status after stroke in squirrel monkeys (Plautz et al., 2003). This improvement appears to be induced by both re-emergence of movement representation in peri-infarct areas as well as the emergence of new areas of representation (Nudo and Milliken, 1996; reviewed in Shin et al., 2014). After repeated stimuli, areas of movement representation have been seen to shift several microns and increase in size with a corresponding increase in spine density in pyramidal cell layers III and V (Nudo et al., 1990; Monfils et al., 2004). Stimulation to other areas of the brain has also gained interest for goals other than motor recovery. For example, deficits in learning and memory in rats after TBI has been improved by theta burst stimulation (TBS) of the fornix and hippocampus (Sweet et al., 2014). There is also human data supporting the use of subthreshold cortical stimulation for recovery after an ischemic infarct (Levy et al., 2008); however, recent data from phase III trials have been negative (Levy et al., 2016).

For patients with severe disorders of consciousness, median nerve stimulation (MNS) has been used to enhance oxygen perfusion to the brain and increase blood brain barrier permeability for medications intended to help stabilize the acute injury environment (Cooper et al., 2006; DeFina et al., 2010). MNS has also been found to increase DA levels, resulting in accelerated wakening from deep coma (Cooper et al., 2006). In 2010, DeFina et al. published an advanced care protocol (ACP) for the rehabilitation of patients in minimally conscious and vegetative states from TBI that involves sequential administration of an array of pharmaceuticals followed by specific interventions and treatments aimed at facilitating neuroplasticity (traditional occupational, physical, and speech therapy plus median nerve stimulation) (DeFina et al., 2010). Patients in this study also finished a 12 week course of pharmaceutical grade nutrients (“neutraceuticals”) which resulted in a modest improvement in disability beyond standard treatment in literature controls.

New closed-loop stimulation (i.e., bio-feedback) paradigms are also being developed for patients who cannot participate in traditional therapy, including brain state dependent stimulation (BSDS) (Gharabaghi et al., 2014a). In 2014, Gharabaghi et al. published an experiment where transcranial magnetic stimulation (TMS) of the motor cortex and haptic feedback to the hand were controlled by sensorimotor desynchronization during motor-imagery in one healthy and one stroke subject with chronic hand paresis (Gharabaghi et al., 2014a). They found that BSDS increased the excitability of the stimulated motor cortex in both patients, an effect not observed in non-BSDS protocols. Both transcranial direct current and magnetic stimulation therapies are in early stages.

Another area of growing interest is robotic therapy, primarily for its ability to deliver highly intense and repetitive motor practice. There is evidence that for some applications, robotic therapy is at least non-inferior to traditional therapy (Reinkensmeyer et al., 2004; Lo et al., 2010; Milot et al., 2013). However, as it stands today, robotic therapy remains limited in its applications, largely because of its inability to individualize goal-directed rehabilitation paradigms suited to unique patient needs. Despite this challenge, robotic interfaces are actively becoming more sophisticated, and a wide range of strategies are now being used to improve whole body functions. For example, an advanced rehabilitative robot with the ability to assess individualized statistics regarding gait and balance stability has been demonstrated on a rat model (Dominici et al., 2012). This particular robot also contains a mode with locomotive capabilities that can go up stairs, as well as a training mode with epidural stimulation, repetitive training, and pharmacologic excitation built in. The latter mode has been shown to enable rats to achieve voluntary over-ground walking, stair walking, and precise paw placement after a stroke. Recently, training for a year with an EEG-based lower extremity exoskeleton and virtual reality leg simulations has been shown to improve somatic sensation and enable new voluntary motor control in the legs of previously chronic complete SCI patients, promoting some patients up to an incomplete paraplegia classification (Donati et al., 2016). As adaptive physiologic mechanisms improve and costs lessen, robotic therapy will likely become increasingly integral to rehabilitative strategies due to its ability to be applied in both a clinic and home setting (Cai et al., 2006; Reinkensmeyer et al., 2006; Courtine et al., 2008).

## Discussion and integration

There are many reasons to be optimistic about the potential for human nervous system repair. For the first time in history we are seeing patients with chronic and complete spinal cord injuries voluntarily move their legs (Dy et al., 2010; Angeli et al., 2014; Gerasimenko et al., 2015b; Donati et al., 2016). Advances in modern neurobiological, neuro-engineering, and neurorehabilitation strategies have provided hope for better outcomes, and the synergistic potential of integrated strategies is only beginning to be realized. In fact, there is a substantial amount of evidence reviewed here that suggests multi-disciplinary approaches might not only be helpful, but will be *critical* for any technique to realize its full therapeutic capability. While the essential nature of many rehabilitation strategies remains a subject of debate, well-timed, goal-directed therapy and some form of associated positive feedback mechanism appear to be necessary components of therapeutic paradigms aimed at generating axonal sprouting and lasting functional improvement (van den Brand et al., 2012; Wahl et al., 2014). This is a profound realization, largely because the abstract concept of how conscious agency relates to neuroanatomical principles of circuitry and guidance remains a mystery (reviewed in Brogaard and Gatzia, 2016; Koch et al., 2016; Sandberg et al., 2016), and data presented in this article suggests that they must be more intimately connected than previously appreciated. Perhaps this relationship can be further interrogated by studying the early development of the nervous system when goal-directed development (e.g., learning to walk or use one's hands) naturally leads to innervation and the establishment of a robust and functional nervous system. On a practical level, the principle of cortically-based intent driving axonal sprouting has at least two important consequences: (1) that patients who suffer from disorders of consciousness (e.g., comatose or vegetative patients) may need completely different therapeutic strategies to attain neural repair than those who have intact consciousness systems, and (2) that experiments which have failed *in vitro* may still be viable therapies when integrated into a macroscopic framework that includes conscious intent and goal-directed therapy with a mechanism for positive feedback.

For patients with disorders of consciousness, perhaps more proximally-based interventions, e.g., cortical stimulation, will find a way to substitute for active participation in rehabilitation. Pairing this stimulation with other feedback mechanisms [e.g., peripheral nerve stimulation and/or passive movement (via exoskeleton, robotic therapy, or physical therapy)] could capitalize on PAS principles discussed in The Neurophysiology Underlying Brain-Machine and Neural Interface Training section. Furthermore, pharmacologic therapy aimed at promoting alertness should be included in treatment strategies.

Another consequence of this theory is that new biological approaches still largely in the *in vitro* stage may need to be applied with goal-directed rehabilitation paradigms to realize their therapeutic potential, and may fail in isolation. Perhaps the clearest support for this claim was the demonstration by Wahl et al. in 2014 of nearly full recovery of skilled forelimb function by rats with large strokes by giving intrathecal injection of an antibody against Nogo-A followed by intensive task-specific training (Wahl et al., 2014). This study demonstrated that sequential application of drug then training was necessary to show benefit, and when immunotherapy and forced-use training were combined simultaneously, functional outcome was worse than either individually. Furthermore, proper combination therapy demonstrated far better results than individual treatments alone. These results serve as evidence that the timing of therapeutic interventions is critical in neurorehabilitation, and that failed treatment strategies pursued in isolation may not, in fact, be futile; instead, they may need to be applied under a different set of circumstances or synergistically with other macro level treatments. This principle could also apply to stem cells (Mothe and Tator, 2012), gene therapies (Warren Olanow et al., 2015), optogenetics (reviewed in Jarvis and Schultz, 2015), neuronal transplantation (Furlanetti et al., 2015), and novel biological or immunotherapies (Maier et al., 2009; Alilain et al., 2011). While details of these treatments remain largely outside the scope of this paper, unfortunately they are all still far from clinical implementation. When ready for rigorous trials, however, evidence presented here suggests that well-timed task-specific physical therapy accompanied by mechanisms like neurostimulation and monoaminergic excitation should be utilized to facilitate their success. Combinations of biological therapies, e.g., stem cell and immunotherapies, may also yield benefit, even if the success of individual applications is found to be modest; in this review, we have shown multiple examples that failure of an individual therapy does not preclude its success in the right context (Benowitz and Yin, 2007; Zai et al., 2011; Wahl et al., 2014). Researchers should also consider delivering local therapies when the extracellular milieu is the most conducive to axonal sprouting and rewiring (i.e., in the first few weeks after injury), as the subacute inflammatory environment may be more conducive to assimilating signals for plasticity and growth than at other times (see The Biology of Neurological Injury and The Physiology of Recovery from Neurological Injury sections) (Shetty and Turner, 1995; Nahmani and Turrigiano, 2014). Since trials for these therapies can carry a very high financial burden and a negative trial may do serious harm to an otherwise potentially viable strategy, the incorporation of the proper macroscopic framework to the application of micro techniques may prove essential for success.

Until complete neurological repair is achievable, optimizing the timing of a variety of treatments and tailoring therapies to different phases of injury and recovery remains the gold standard approach (Kleim and Jones, 2008; Pekna et al., 2012). In American hospitals, standard of care during the acute phase of neurological injury aims to stabilize the injury and prevent further loss of tissue through (1) the initiation of hypothermia after cardiac arrest, (2) the maintenance of neural perfusion pressures after TBI or SCI, (3) the recannulation of occluded vessel(s) after stroke by thrombectomy or thrombolysis, or (4) surgery to evacuate mass lesions or decompress edematous tissue. Once stabilized, however, when and how best to apply pharmaceutical, rehabilitation, and neurostimulation strategies to aid in neural repair needs rigorous study (reviewed in Lang et al., 2015). While certain biological manipulations discussed in previous sections have shown promise in the lab (e.g., multiple NgR deletions, anti-NogoA antibodies, ionosine application, CAST/Ei knockouts), translation to bedside applications remains a challenge. Furthermore, generating axonal regrowth on its own does not ensure restoration of function (see Molecular Mechanisms of Neural Repair section). Also notable in clinical applications is the relative paucity of biomarkers that might help drive prognosis and diagnose phases of injury/recovery, as well as the large discrepancy between known mechanisms of injury-repair and viable options for intervention (reviewed in Hergenroeder et al., 2008; DeFina et al., 2009; Zetterberg et al., 2013). Now that outcomes may start improving, it would be interesting to monitor known markers through improved recovery periods, as well as to search for new ones in the hopes of helping inform the timing and selection of therapeutic applications. Perhaps by combining new developments in the neurobiology lab with the essential elements of neurological rehabilitation, neural simulation, and the concept of multi-disciplinary intervention on micro- and macroscopic levels, success will be found where previously promising therapies have failed (Alilain et al., 2011; Warren Olanow et al., 2015; Levy et al., 2016).

## Conclusion

Researchers have long been developing ways to improve the quality of life for patients who suffer from SCI, stroke, and other neurological disorders classically categorized as permanent. Several disciplines, namely neurobiology, neuro-engineering, and neurorehabilitation, have all made great strides. However, the path to achieving complete neurologic recovery for human patients remains remote and complex. Nonetheless, patients are now starting to show recovery beyond that which was previously thought possible. This paper examined the most recent advances in the biology of neurological injury, molecular mechanisms of neural repair, physiology of neurological recovery, neurophysiology underlying brain-machine and neural interface training, state of the art in neural and brain-machine interfaces, neurorehabilitation strategies, and ideas for how to integrate future research. Furthermore, we have identified key elements of repair strategies that should be included in these studies. As the development of immunotherapies, electrical stimulation, neural interfaces, stem cells, optogenetics, and gene therapies advance, their reparative potential may only be realized by integrating them into a rehabilitation framework that includes conscious intention and positive neural feedback. Special attention should be paid to timing, sequence, and dose of therapy. Hopefully, these concepts will help usher in the next frontier of nervous system recovery.

## Author contributions

MK, SR, MS, VE, and DT all contributed substantially to the intellectual property of this project. Each author either drafted or revised the document critically multiple times, and each has given his final approval of the version to be published. Furthermore, each author agrees to be accountable for all aspects of the work.

## Funding

MK is supported by a grant from the National Institute of Neurological Disorders and Stroke (NINDS; R25, 5R25NS065731-08). MS is supported by grants from the National Institute of Health (NIH; K08NS060223 and R01NS094748), Paralyzed Veterans of America, Brain Research Foundation, Doris Duke Charitable Foundation, and Northwestern Memorial Foundation (Dixon Translational Research Grant). VE is supported by a grants from the National Institute of Biomedical Imaging and Bioengineering (NIBIB; U01EB007615, U01EB015521, R43EB017641, and R43EB018232), Paralyzed Veterans of America, WalkAbout Foundation, Christopher & Dana Reeve Foundation, and Broccoli Foundation. DT is supported by grants from the NIH (R21, AG051103; RO1, NS079312; R21, NS084176; and R37, NS040894) and Veteran's Affairs (VA; VA I21, BX003023; and VA I21, RX002223).

### Conflict of interest statement

The authors declare that the research was conducted in the absence of any commercial or financial relationships that could be construed as a potential conflict of interest. The reviewer BN and handling Editor declared their shared affiliation, and the handling Editor states that the process nevertheless met the standards of a fair and objective review.
